# INTEREST GROUP SESSION—CHINESE GERONTOLOGY STUDIES: DEVELOPING A BODY-MIND-SPIRIT CAREGIVER SELF-MANAGEMENT PROGRAM AND A MOBILE APPLICATION FOR CHINESE AMERICANS

**DOI:** 10.1093/geroni/igz038.2010

**Published:** 2019-11-08

**Authors:** Iris Chi, Walter Boot

**Affiliations:** 1 University of Southern California, Los Angeles, California, United States; 2 Florida State University, Tallahassee, Florida, United States

## Abstract

Caregivers of older adults are at risk for poor physical and mental health outcomes. Chinese American caregivers, especially those who are recent immigrants, are at risks for developing health symptoms while less likely to seek outside help and utilize caregiver resources due to cultural barriers, low health literacy, and limited-English proficiency. This symposium will introduce the Caregiver Self-Management Program (CSMP), a training program based on the Body-Mind-Spirit model to equip Chinese immigrant caregivers with self-care knowledge, skills, and self-efficacy. We designed the in-person training to be four 3-hour sessions and pilot tested it among 11 Chinese caregivers. The training was well-received by participants; however, the caregivers’ duties and their working status, compounded with transportation barriers, leave them little time to participate in the intervention. To address these challenges, we further employed a user participatory approach to design, prototype, and pilot-test a CSMP mobile application (app) to meet the needs of Chinese immigrant caregivers. App functions included interactive multi-media lessons and practice, scheduling of coaching sessions for problem solving, extended readings, community resources, etc. We co-designed the app with seven caregiver co-designers, alpha-tested the app prototype with 12 caregivers, and beta-tested the app in real-life settings with 20 caregivers. Four presentations in the symposium will discuss the CSMP in-person training design and evaluation, influence of Chinese culture on caregiving and impacts of CSMP, user-participatory approach used in co-design and alpha-testing of the app prototype, and preliminary results from the beta-testing to assess external user acceptance and feasibility of the CSMP app.

